# Recent hospitalization for Non-coronary events and use of preventive medications for coronary artery disease: An observational cohort study

**DOI:** 10.1186/1471-2261-11-42

**Published:** 2011-07-09

**Authors:** Steven M Bradley, Chris L Bryson, Charles Maynard, Thomas M Maddox, Stephan D Fihn

**Affiliations:** 1 Health Services Research & Development Northwest Center of Excellence, Veterans Affairs Puget Sound Health Care System, and University of Washington, Seattle, WA, USA; 2 VA Eastern Colorado Health Care System and University of Colorado Denver, Denver, CO, USA

## Abstract

**Background:**

High-quality systems have adopted a comprehensive approach to preventive care instead of diagnosis or procedure driven care. The current emphasis on prescribing medications to prevent complications of coronary artery disease (CAD) at discharge following an acute coronary syndrome (ACS) may exclude high-risk patients who are hospitalized with conditions other than ACS.

**Methods:**

Among a sample of patients with CAD treated at Veterans Affairs medical centers between January, 2005 and November, 2006, we investigated whether recent non-ACS hospitalization was associated with prescriptions of preventive medications as compared with patients recently hospitalized with ACS.

**Results:**

Of 13,211 patients with CAD, 58% received aspirin, 70% β-blocker, 60% angiotensin-converting enzyme inhibitor (ACE-I) or angiotensin II receptor blocker (ARB), and 65% lipid-lowering therapy. Twenty-five percent of eligible patients were receiving all four medications. Having been hospitalized for a non-ACS event in the prior 6 months did not substantially affect the adjusted proportion on preventive medications. In contrast, among patients hospitalized for ACS in the prior 6 months, the adjusted proportion prescribed aspirin was 21% higher (p < 0.001), β-blocker was 14% higher (p < 0.001), ACE-I or ARB was 9% higher (p < 0.001), lipid therapy was 12% higher (p < 0.001), and prescribed all four medications was 18% higher (p < 0.001) than among patients hospitalized for ACS more than 2 years earlier.

**Conclusions:**

Being hospitalized for a non-ACS condition did not appear to influence preventive medication use among patients with CAD and represents a missed opportunity to improve patient care. The same protocols employed to improve use of preventive medications in patients discharged for ACS might be extended to CAD patients discharged for other conditions as well.

## Background

Strategies to improve provision of medications effective in preventing complications of coronary artery disease (CAD) have focused largely on patients who have just experienced an acute coronary event [1-3]. Although these patients represent a high risk group and ensuring that they receive preventive medications at discharge after an acute coronary syndrome (ACS) is important, this strategy overlooks other opportunities that systems and providers have to improve care for the larger group of patients with CAD who are not experiencing ACS. For example, it is unknown if patients hospitalized because of an illness other than ACS have their cardiovascular medications appropriately adjusted prior to discharge.

We sought to determine if recent non-ACS hospitalization was associated with use of preventive cardiac medications in comparison with patients admitted with ACS. Given the emphasis on secondary prevention measures for CAD at the time of discharge for ACS and prior studies demonstrating high rates of preventive medication prescription at discharge for ACS [1-5], we anticipated a large effect of recent ACS hospitalization on the proportion of patients on preventive medications. Comparatively, we hypothesized recent non-ACS hospitalization would have a minimal impact on the proportion of patients with coronary disease on preventive medications and represent a previously unappreciated opportunity to improve CAD preventive care.

## Methods

Data from our study were obtained from the Cardiac Care Follow-Up Clinical Study (CCFCS) which is derived from the External Peer Review Program for quality monitoring of acute myocardial infarction and unstable angina in the Veterans Health Administration. This program includes data on 100% of patients diagnosed with acute myocardial infarction and a random sample of 10% of patients diagnosed with unstable angina treated at VA medical centers during the study period. Patients were identified by *International Classification of Diseases, Ninth Revision (ICD-9)*, diagnosis codes 410.xx (myocardial infarction) and 411.xx (unstable angina) from administrative data. Resulting patient lists were then transmitted to Veterans Affairs (VA) facilities, where both paper and electronic medical records were manually abstracted by trained abstractors using standard reporting forms into the CCFCS data repository. The study was approved by the Institutional Review Board of VA Puget Sound. Additional details regarding CCFCS have been described previously [6].

From patients in CCFCS admitted between January 1, 2005 and November 22, 2006, we identified patients with a prior history of myocardial infarction, unstable angina, or coronary atherosclerosis as determined by review of ICD-9 diagnosis codes (410.xx, 411.xx, 412.xx, or 414.xx) for all inpatient and outpatient visits in the 3 years prior to the admission date. Thus, all patients within the study cohort had a history of CAD within the 3 years preceding the ACS hospitalization captured by CCFCS. For patients with multiple hospitalizations captured within the CCFCS study period, we considered the most recent hospitalization as the index event. We excluded patients with missing data regarding outpatient medications for coronary prevention at the time of index admission. Patients with an absolute (i.e. medication allergy) or relative contraindication to a medication were excluded from analyses of that medication. Relative contraindications for aspirin included use of warfarin prior to admission, history of anemia, or history of ulcer. History of renal failure was considered a relative contraindication to angiotensin-converting enzyme inhibitor (ACE-I) or angiotensin II receptor blocker (ARB) and history of liver disease was considered a relative contraindication to use of lipid lowering medications.

The main outcomes of interest were receipt of outpatient prescriptions for preventive medications (aspirin, β-blocker, lipid-lowering medication, and ACE-I or ARB) as determined from chart review of the admission records at the time of index hospitalization for ACS in the CCFCS registry. The exposures of interest were the time interval from prior non-ACS and ACS hospitalizations in the VA to the index event. For each patient, a time since prior non-ACS hospitalization and time since prior ACS hospitalization was determined separately. These intervals were determined by review of all discharges from VA health care in the two years preceding the index event and measured from the date of discharge to the date of admission for the index event. Previous hospitalizations were determined to have been for ACS if the primary diagnosis code was myocardial infarction, unstable angina, or coronary atherosclerosis (ICD-9 of 410.XX, 411.XX, 412.XX, or 414.XX) [7]. When the discharge date for the previous hospitalization and index admission date were the same, as in the case of patient transfer from another facility, the preceding hospitalization was considered for this interval. We grouped these intervals into ≤ 6 months, 6-12 months, 12-24 months, and > 24 months since last hospitalized. These intervals were chosen to maximize sample size in the comparison intervals prior to 2 years.

We used data from the CCFCS data registry to describe demographic and personal characteristics (i.e. age, sex, current smoker, hypertension, diabetes, heart failure, cerebral vascular disease, peripheral vascular disease, chronic kidney disease, and chronic obstructive pulmonary disease). Ethnicity was based on a combination of self-report and the electronic record [8]. Socioeconomic status was based on the VA means test [9].

We present unadjusted descriptive summaries of baseline patient characteristics for the study cohort. We report unadjusted descriptive statistics of the proportion of eligible patients on individual medications and multiple medications by patient characteristics. We conducted analyses separately for each individual medication and cumulative medications of interest as the eligible population varied due to contraindications to therapies.

We anticipated missing values for our ethnicity covariate [8]. Instead of ignoring missing data using complete-case analysis, we conservatively chose to impute missing covariate values with chained equations. This method provides less biased estimates than complete-case analysis [10]. From the imputed data, multivariate logistic regression estimated the effect size of time interval since hospitalization for non-ACS and ACS episodes on the prescription of preventive medications prior to recurrent event after adjustment for other covariates [11]. Our model included both exposures of interest and the following covariates: age (< 55, 55-65, 65-75, ≥ 75 as indicator variables), sex, ethnicity, socioeconomic status (low, middle, high as indicator variables), current smoker, hypertension, diabetes, heart failure, cerebral vascular disease, peripheral vascular disease, chronic kidney disease, and chronic obstructive pulmonary disease. We did not perform hypothesis testing to determine patient characteristics included in the regression analyses, but instead included standard covariates that have been associated with preventive medication use in a prior analysis [12]. We also included age and socioeconomic status as indicator variables to allow for non-linear associations between these variables and preventive medication use. From our logistic regression results, we report the proportion of patients prescribed medications for the exposure of interest adjusted to reflect the mean for each covariate in the cohort. This was determined for each level of the predictor of interest by calculation of the log odds and standard error within each imputed dataset, combination of log odds and standard errors across datasets using methods described by Rubin,[13] and transformation of log odds and standard errors to proportions and confidence intervals. We report adjusted proportions or percentages in place of odds ratios as our outcome was common and odds ratios do not approximate relative risks in this setting [14,15].

We conducted a number of sensitivity analyses to evaluate the robustness of our findings. First, we repeated our analyses after excluding patients with a history of dementia or cancer as providers may have anticipated less benefit to preventive therapies in this setting. We then repeated our analyses after stratifying on past history of myocardial infarction. This stratified analysis was to ensure any observed effect size in the primary analysis was not related to inclusion of low-risk patients with a remote history of ACS. We next explored the potential for bias related to exclusion of patients with incomplete ascertainment of preventive medication history. Finally, we completed a complete-case analysis to estimate the influence of imputation on our results. All statistical analyses were conducted with Stata, version 10.0 (StatCorp LP, College Station, TX). All hypotheses were evaluated at a two-sided significance level of 0.05, with calculation of 95% confidence intervals.

## Results

**Figure 1:**
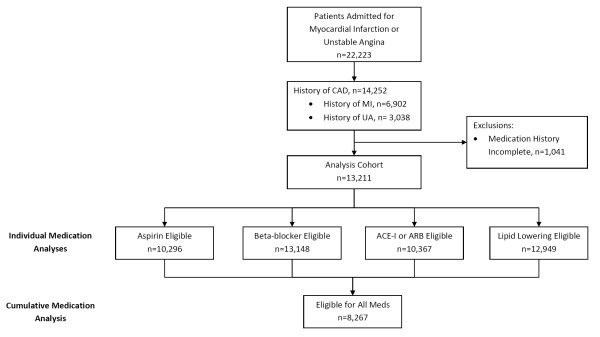
**Identification of Study Cohort**. CAD = coronary artery disease; ACE-I = angiotensin-converting enzyme inhibitor, ARB = angiotensin II receptor blocker, Meds = medications.

There were 22,223 patients admitted for ACS during the study period, of whom, 14,252 (64%) had a diagnosis of CAD within the previous 3 years. We excluded 1,041 (7%) patients whose medications were not assessed at the time of admission. After exclusion of patients with absolute or relative contraindications, 10,296 (78%) patients were deemed eligible to receive aspirin, 13,148 (> 99%) β-blockers, 10,367 (78%) ACE-I or ARB, 12,949 lipid-lowering therapy, and 8,267 (63%) were eligible for all four medications (Figure 1).

The patients studied were largely elderly, white men (Table 1). Ethnicity was missing for 25% of patients as anticipated from prior studies using similar VA data sources [8]. Data were complete for all other covariates. Nearly 50% of the cohort had been hospitalized for a non-ACS event in the previous 2 years, while less than 25% had been hospitalized for ACS during that time frame.

**Table 1:** Baseline Patient Characteristics of Analysis Cohort

Characteristic	Entire Cohort (n = 13,211)
Age (yrs)	
< 55	1,162 (8.8%)
55-65	3,900 (29.5%)
65-75	3,209 (24.3%)
≥ 75	4,940 (37.4%)
Female	169 (1.3%)
Ethnicity	
White	7,461 (56.5%)
Non-white	2,105 (15.9%)
Unknown	3,645 (27.6%)
Socioeconomic status	
Low	6,875 (52.0%)
Middle	4,917 (37.2%)
High	1,419 (10.7%)
Current smoker	3,190 (24.2%)
Coronary artery bypass grafting	4,087 (30.9%)
Percutaneous coronary intervention	3,042 (23.0%)
Hypertension	9,360 (70.9%)
Hyperlipidemia	9,512 (72.0%)
Diabetes	2,373 (18.0%)
Heart failure	5,513 (41.7%)
Cerebral vascular disease	1,019 (7.7%)
Peripheral vascular disease	2,028 (15.4%)
Chronic kidney disease	2,398 (18.2%)
Chronic obstructive pulmonary disease	2,515 (19.0%)
Time interval since ACS hospitalization	
≥ 6 months	1,623 (12.3%)
6-12 months	616 (4.7%)
12-24 months	779 (5.9%)
> 24 months	10,193 (77.2%)
Time interval since non-ACS hospitalization	
≤ 6 months	3,460 (26.2%)
6-12 months	1,248 (9.5%)
12-24 months	1,343 (10.2%)
> 24 months	7,160 (54.2%)

Data was complete for all covariates with the exception of ethnicity.

ACS = acute coronary syndrome.

β-blockers were the most commonly prescribed preventive medication (70% of eligible patients), while lipid-lowering therapy was prescribed in nearly 65%, ACE-I or ARB in 60%, and aspirin in 58% of eligible patients (Table 2). Only 25% of patients eligible for all four medications were prescribed the full combination of preventive therapies. A higher proportion of patients were on all four medications if they had a prior history of revascularization by PCI or CABG, or if they had a history of hypertension, hyperlipidemia, diabetes, or heart failure.

**Table 2:** Proportion of Eligible Patients on Preventive Medications by Baseline Characteristics

Characteristic	Prescribed Medication, % (n = Eligible Patients)				
	
	Aspirin (n = 10,296)	B-blocker (n = 13,148)	ACE-I/ARB (n = 10,367)	Lipid lowering (n = 12,949)	All Meds (n = 8,267)
All eligible patients	58.3	70.2	60.3	64.8	25.0
Age (yrs)					
< 55	58.0	65.6	54.3	61.5	23.5
55-65	59.3	70.3	60.4	66.8	28.3
65-75	56.2	73.9	64.6	69.2	25.7
≥ 75	59.0	68.8	58.9	61.2	21.3
Female	57.6	67.3	60.5	63.3	20.2
Ethnicity					
White	60.1	71.7	60.9	66.2	26.1
Non-white	60.9	69.3	63.6	62.8	25.7
Unknown	53.5	67.7	57.3	63.0	22.6
Socioeconomic status					
Low	57.2	70.3	60.8	63.9	24.4
Middle	62.1	70.9	60.3	65.0	26.5
High	50.9	67.3	57.4	68.0	22.5
Current smoker	56.3	66.4	55.9	63.0	23.8
CABG	63.3	79.1	66.5	75.0	32.8
PCI	65.3	77.2	65.4	72.9	32.4
Hypertension	59.1	72.1	64.4	66.7	26.6
Hyperlipidemia	59.7	72.7	62.6	70.6	27.4
Diabetes	62.4	76.3	70.7	68.8	29.8
Heart failure	61.9	76.8	70.0	67.3	30.9
Cerebral vascular disease	59.1	71.2	63.3	66.0	24.5
Peripheral vascular disease	61.1	73.4	64.3	67.5	25.9
Chronic kidney disease	59.2	76.4	60.9	67.5	27.4
COPD	57.7	67.2	59.7	63.5	22.7

Data was complete for all covariates with the exception of ethnicity. ACE-I = angiotensin-converting enzyme inhibitor; ARB = angiotensin II receptor blocker; CABG = coronary artery bypass grafting; PCI = percutaneous coronary intervention; COPD = chronic obstructive pulmonary disease.

**Figure 2:**
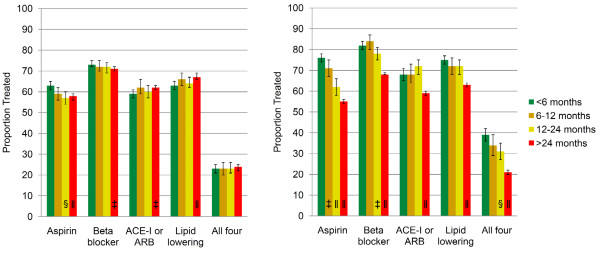
**Adjusted Proportion on Preventive Medications by Time Interval Since Prior Hospitalization**. **A**. Adjusted Proportion on Medications by Time Interval Since Last ACS Hospitalization* **B**. Adjusted Proportion on Medications by Time Interval Since Last N-ACS Hospitalization^†^. *Adjusted for age, sex, race, socioeconomic status, current smoker, hypertension, diabetes, congestive heart failure, cerebral vascular disease, peripheral vascular disease, chronic kidney disease, and time since non-ACS hospitalization. † Adjusted for covariates as above with time since ACS hospitalization in place of time since non-ACS hospitalization. ‡ p < 0.05 compared to < 6 months. § p < 0.01 compared to < 6 months. ‖ p < 0.001 compared to < 6 months.

The adjusted proportion of eligible patients on preventive medications by time since last hospitalization is shown in Figure 2, stratified by time since prior hospitalization for non-ACS (Figure 2A) and ACS hospitalization (Figure 2B). The adjusted proportion of eligible patients on secondary preventive medications was influenced only minimally by recent non-ACS hospitalization. Among patients hospitalized during the past 6 months for a non-ACS event, the proportion prescribed aspirin was 5% higher (p < 0.001), β-blockers was 2% (p = 0.02) higher, the proportion on ACE-I or ARB was 3% (p = 0.04) lower, on lipid-lowering therapy was 4% (p < 0.001) lower, and on all four medications was 1% higher (p = 0.86) compared with patients not hospitalized for a non-ACS event in the past 2 years.

Recent hospitalization for ACS was associated with a higher likelihood of receiving preventive medications. The adjusted proportion on aspirin was 21% higher (p < 0.001), β-blockers was 14% higher (p < 0.001), on ACE-I or ARB was 9% higher (p < 0.001), on lipid-lowering therapy was 12% higher (p < 0.001), and on all four medications was 18% higher (p < 0.001) compared with patients hospitalized for ACS more than 2 years ago (Table 3).

**Table 3:** Proportion on Preventive Medications by Time Interval Since Last Hospitalized

	Medication (n = Eligible Patients)									
	Aspirin (n = 10,296)		β**-blocker** (n = 13,148)		ACE-I/ARB (n = 10,367)		Lipid Lowering (n = 12,949)		All Meds (n = 8,267)	
**Time Interval Since** **ACS Hospitalization**	**Unadjusted Proportion (95% CI)**	Adjusted Proportion (95% CI)*	**Unadjusted Proportion (95% CI)**	Adjusted Proportion (95% CI)*	**Unadjusted Proportion (95% CI)**	Adjusted Proportion (95% CI)*	**Unadjusted Proportion (95% CI)**	Adjusted Proportion (95% CI)*	**Unadjusted Proportion (95% CI)**	Adjusted Proportion (95% CI)*
**< 6 months**	77 (75-80)	76 (74-79)	83 (81-85)	82 (80-84)	70 (67-73)	68 (66-71)	75 (73-77)	75 (73-77)	42 (39-45)	39 (36-42)
**6-12 months**	73 (69-77)	71 (66-75)^‡^	86 (83-88)	84 (80-87)	70 (66-74)	68 (63-72)	74 (70-77)	72 (68-76)	39 (34-44)	34 (29-39)
**12-24 months**	64 (60-68)^||^	62 (58-66)^||^	80 (77-83)	78 (75-81)^‡^	74 (70-77)	72 (68-75)	74 (71-77)	72 (69-75)	36 (31-40)^‡^	31 (27-35)^§^
**> 24 months**	54 (53-55)^||^	55 (54-56)^||^	66 (66-67)^||^	68 (68-69)^||^	57 (56-58)^||^	59 (58-60)^||^	62 (61-63)^||^	63 (62-64)^||^	21 (20-22)^||^	21 (20-22)^||^

**Time Interval Since** **Non-ACS Hospitalization**	**Unadjusted Proportion (95% CI)**	**Adjusted Proportion (95% CI)^†^**	**Unadjusted Proportion (95% CI)**	**Adjusted Proportion (95% CI)^†^**	**Unadjusted Proportion (95% CI)**	**Adjusted Proportion (95% CI)^†^**	**Unadjusted Proportion (95% CI)**	**Adjusted Proportion (95% CI)^†^**	**Unadjusted Proportion (95% CI)**	**Adjusted Proportion (95% CI)^†^**
**< 6 months**	63 (62-65)	63 (61-65)	73 (72-75)	73 (72-75)	61 (59-62)	59 (57-61)	62 (60-64)	63 (61-65)	26 (24-28)	23 (21-25)
**6-12 months**	60 (57-63)	59 (56-62)	73 (70-75)	72 (70-75)	64 (61-67)	62 (59-66)	65 (63-68)^‡^	66 (63-69)	26 (23-29)	23 (20-26)
**12-24 months**	57 (54-60)^||^	57 (54-60)^§^	71 (69-73)	72 (69-74)	60 (57-63)	60 (57-63)	64 (61-66)	64 (62-67)	26 (23-29)	23 (21-27)
**> 24 months**	56 (55-57)^||^	58 (56-59)^||^	68 (67-69)^||^	71 (70-72)^‡^	60 (58-61)	62 (61-63)^‡^	66 (65-67)^||^	67 (66-69)^||^	24 (23-26)	24 (22-25)

*Adjusted for age, sex, race, socioeconomic status, current smoker, hypertension, diabetes, congestive heart failure, cerebral vascular disease, peripheral vascular disease, chronic kidney disease, and time since non-ACS hospitalization.

†Adjusted for covariates as above with time since last ACS hospitalization in place of time since non-ACS hospitalization.

‡ p < 0.05 compared to < 6 months.

§ p < 0.01 compared to < 6 months.

|| p < 0.001 compared to < 6 months.

In sensitivity analyses, excluding patients with a history of dementia (n = 2,291) or cancer (n = 926) increased the total proportion of patients prescribed each medication by approximately 1%, but no appreciable change was noted in the proportion of patients on preventive medications by time since last non-ACS or ACS hospitalization (Table 4). Similarly, stratification on history of myocardial infarction did not substantially change the results (Table 5). In our study, we intentionally limited our analysis time frame to the period that included data on ARB use within CCFCS to minimize misclassification of ACE-I or ARB use as an outcome. Despite this restriction, missingness on ARB use constituted 997 (> 99%) of the patients excluded in our analysis. Our findings were unchanged in an analysis of preventive medications other than ACE-I or ARB in the expanded cohort that included patients with missingness on ARB use. Finally, our complete-case analysis suggested our results were minimally influenced by our imputation technique (Table 6).

**Table 4:** Proportion on Preventive Medications by Time Interval Since Last Hospitalized After Exclusion of Patients with History of Dementia or Cancer

	Medication (n = Eligible Patients)									
	Aspirin (n = 7,984)		β**-blocker** (n = 9,941)		ACE-I/ARB (n = 7,876)		Lipid Lowering (n = 9,824)		All Meds (n = 6,452)	
**Time Interval Since** **ACS Hospitalization**	**Unadjusted Proportion (95% CI)**	Adjusted Proportion (95% CI)*	**Unadjusted Proportion (95% CI)**	Adjusted Proportion (95% CI)*	**Unadjusted Proportion (95% CI)**	Adjusted Proportion (95% CI)*	**Unadjusted Proportion (95% CI)**	Adjusted Proportion (95% CI)*	**Unadjusted Proportion (95% CI)**	Adjusted Proportion (95% CI)*
**< 6 months**	77 (75-80)	76 (73-79)	84 (82-86)	83 (81-85)	71 (68-74)	70 (66-73)	75 (73-78)	75 (73-78)	44 (40-47)	40 (37-44)
**6-12 months**	73 (68-77)	71 (66-75)	86 (83-89)	84 (80-87)	72 (67-76)	69 (64-74)	74 (70-78)	73 (68-77)	39 (34-45)	35 (29-41)
**12-24 months**	64 (60-68)^||^	62 (57-66)^||^	80 (77-83)	78 (74-81)^‡^	76 (71-80)	74 (69-78)	74 (70-78)	72 (68-76)	35 (30-40)^§^	30 (26-35)^§^
**> 24 months**	54 (53-55)^||^	55 (53-56)^||^	68 (67-69)^||^	70 (69-71)^||^	58 (57-60)^||^	60 (59-61)^||^	64 (63-65)^||^	66 (65-67)^||^	22 (20-23)^||^	21 (20-22)^||^

**Time Interval Since** **Non-ACS Hospitalization**	**Unadjusted Proportion (95% CI)**	**Adjusted Proportion (95% CI)^†^**	**Unadjusted Proportion (95% CI)**	**Adjusted Proportion (95% CI)^†^**	**Unadjusted Proportion (95% CI)**	**Adjusted Proportion (95% CI)^†^**	**Unadjusted Proportion (95% CI)**	**Adjusted Proportion (95% CI)^†^**	**Unadjusted Proportion (95% CI)**	**Adjusted Proportion (95% CI)^†^**
**< 6 months**	65 (63-67)	64 (61-67)	76 (74-77)	75 (73-77)	63 (60-65)	60 (58-63)	64 (62-66)	65 (63-67)	28 (25-31)	25 (22-27)
**6-12 months**	60 (57-64)^‡^	59 (55-63)^‡^	76 (73-78)	75 (71-78)	67 (63-70)	64 (60-68)	69 (66-72)^‡^	69 (65-72)	27 (23-31)	23 (19-27)
**12-24 months**	57 (53-61)^||^	56 (53-60)^§^	74 (71-76)	74 (71-77)	63 (59-66)	62 (58-66)	66 (63-69)	67 (63-70)	27 (23-31)	24 (21-28)
**> 24 months**	56 (55-57)^||^	57 (56-59)^||^	69 (67-70)^||^	72 (70-73)^§^	60 (59-61)	63 (61-64)	67 (66-68)^‡^	69 (67-70)^‡^	25 (23-26)^‡^	24 (23-26)

*Adjusted for age, sex, race, socioeconomic status, current smoker, hypertension, diabetes, congestive heart failure, cerebral vascular disease, peripheral vascular disease, chronic kidney disease, and time since non-ACS hospitalization.

†Adjusted for covariates as above with time since last ACS hospitalization in place of time since non-ACS hospitalization.

‡ p < 0.05 compared to < 6 months.

§ p < 0.01 compared to < 6 months.

|| p < 0.001 compared to < 6 months.

**Table 5:** Proportion on Preventive Medications by Time Interval Since Last Hospitalized Limited to Patients with Past History of Myocardial Infarction After Exclusion of Patients with History of Dementia or Cancer

	Medication (n = Eligible Patients)									
	Aspirin (n = 4,691)		β**-blocker** (n = 5,830)		ACE-I/ARB (n = 4,603)		Lipid Lowering (n = 5,757)		All Meds (n = 3,794)	
**Time Interval Since** **ACS Hospitalization**	**Unadjusted Proportion (95% CI)**	Adjusted Proportion (95% CI)*	**Unadjusted Proportion (95% CI)**	Adjusted Proportion (95% CI)*	**Unadjusted Proportion (95% CI)**	Adjusted Proportion (95% CI)*	**Unadjusted Proportion (95% CI)**	Adjusted Proportion (95% CI)*	**Unadjusted Proportion (95% CI)**	Adjusted Proportion (95% CI)*
**< 6 months**	79 (76-81)	77 (74-80)	84 (82-86)	83 (81-86)	71 (67-74)	69 (66-73)	75 (72-78)	75 (72-78)	43 (39-47)	39 (35-44)
**6-12 months**	72 (67-77)^‡^	70 (64-75)^‡^	87 (83-90)	86 (81-89)	72 (66-77)	69 (63-75)	74 (69-78)	72 (66-77)	40 (33-47)	35 (29-42)
**12-24 months**	65 (60-70)^||^	63 (57-68)^||^	81 (76-84)	78 (73-82)^‡^	74 (69-79)	72 (66-77)	72 (67-76)	70 (64-74)	37 (31-43)	33 (27-39)
**> 24 months**	55 (54-57)^||^	57 (55-58)^||^	67 (65-68)^||^	70 (68-71)^||^	58 (57-60)^||^	60 (58-62)^||^	63 (62-65)^||^	65 (64-67)^||^	23 (21-24)^||^	23 (21-24)^||^

**Time Interval Since** **Non-ACS Hospitalization**	**Unadjusted Proportion (95% CI)**	**Adjusted Proportion (95% CI)^†^**	**Unadjusted Proportion (95% CI)**	**Adjusted Proportion (95% CI)^†^**	**Unadjusted Proportion (95% CI)**	**Adjusted Proportion (95% CI)^†^**	**Unadjusted Proportion (95% CI)**	**Adjusted Proportion (95% CI)^†^**	**Unadjusted Proportion (95% CI)**	**Adjusted Proportion (95% CI)^†^**
**< 6 months**	70 (67-73)	68 (65-71)	78 (76-80)	77 (75-80)	64 (60-67)	61 (57-64)	66 (64-69)	67 (64-70)	33 (29-37)	29 (25-32)
**6-12 months**	62 (57-66)^§^	59 (54-64)^§^	76 (72-80)	75 (70-78)	65 (60-70)	61 (55-66)	69 (65-73)	69 (64-73)	29 (24-34)	24 (19-29)
**12-24 months**	60 (55-64)^||^	58 (54-63)^§^	75 (71-78)	75 (71-78)	66 (62-71)	65 (60-70)	66 (62-70)	65 (61-69)	31 (26-36)	26 (22-31)
**> 24 months**	58 (56-60)^||^	60 (58-62)^||^	68 (67-70)^||^	73 (71-74)^§^	60 (58-62)	63 (61-65)	66 (64-67)	68 (66-69)	26 (24-27)^||^	26 (24-27)

*Adjusted for age, sex, race, socioeconomic status, current smoker, hypertension, diabetes, congestive heart failure, cerebral vascular disease, peripheral vascular disease, chronic kidney disease, and time since non-ACS hospitalization.

†Adjusted for covariates as above with time since last ACS hospitalization in place of time since non-ACS hospitalization.

‡ p < 0.05 compared to < 6 months.

§ p < 0.01 compared to < 6 months.

|| p < 0.001 compared to < 6 months.

**Table 6:** Complete-case Analysis of Proportion on Preventive Medications by Time Interval Since Last Hospitalized

	Medication (n = Eligible Patients)									
	Aspirin (n = 7,311)		β**-blocker** (n = 9,524)		ACE-I/ARB (n = 7,383)		Lipid Lowering (n = 9,370)		All Meds (n = 8,267)	
**Time Interval Since** **ACS Hospitalization**	**Unadjusted Proportion (95% CI)**	Adjusted Proportion (95% CI)*	**Unadjusted Proportion (95% CI)**	Adjusted Proportion (95% CI)*	**Unadjusted Proportion (95% CI)**	Adjusted Proportion (95% CI)*	**Unadjusted Proportion (95% CI)**	Adjusted Proportion (95% CI)*	**Unadjusted Proportion (95% CI)**	Adjusted Proportion (95% CI)*
**< 6 months**	78 (75-81)	77 (74-80)	82 (80-84)	82 (79-84)	69 (66-72)	68 (64-71)	74 (72-77)	74 (72-77)	41 (38-45)	38 (34-42)
**6-12 months**	73 (68-78)	71 (66-76)^‡^	85 (82-88)	84 (80-87)	71 (66-76)	70 (64-74)	74 (70-78)	73 (68-77)	39 (33-45)	35 (29-41)
**12-24 months**	66 (62-71)^||^	65 (60-69)^||^	81 (77-84)	79 (75-82)	75 (71-79)^‡^	74 (69-78)^‡^	74 (70-77)	73 (69-76)	38 (33-43)	34 (29-39)
**> 24 months**	56 (55-57)^||^	57 (56-58)^||^	68 (67-69)^||^	70 (68-71)^||^	59 (57-60)^||^	60 (59-61)^||^	63 (62-64)^||^	64 (63-65)^||^	22 (21-23)^||^	22 (20-23)^||^

**Time Interval Since** **Non-ACS Hospitalization**	**Unadjusted Proportion (95% CI)**	**Adjusted Proportion (95% CI)^†^**	**Unadjusted Proportion (95% CI)**	**Adjusted Proportion (95% CI)^†^**	**Unadjusted Proportion (95% CI)**	**Adjusted Proportion (95% CI)^†^**	**Unadjusted Proportion (95% CI)**	**Adjusted Proportion (95% CI)^†^**	**Unadjusted Proportion (95% CI)**	**Adjusted Proportion (95% CI)^†^**
**< 6 months**	65 (62-67)	64 (62-66)	73 (71-74)	73 (71-75)	60 (58-63)	59 (57-62)	61 (60-63)	63 (61-65)	25 (23-28)	23 (21-25)
**6-12 months**	61 (57-64)	61 (57-64)	73 (70-75)	73 (70-76)	64 (60-67)	63 (59-67)	66 (63-69)^§^	67 (64-70)^‡^	26 (23-30)	24 (20-27)
**12-24 months**	59 (55-62)^§^	59 (56-62)^‡^	71 (68-74)	72 (69-75)	61 (58-64)	61 (58-65)	63 (60-66)	64 (61-67)	27 (24-30)	25 (21-28)
**> 24 months**	58 (57-60)^||^	60 (58-61)^§^	70 (69-71)^‡^	73 (71-74)	62 (60-63)	64 (62-65)^§^	68 (67-69)^||^	69 (67-70)^||^	26 (25-28)	25 (24-27)

*Adjusted for age, sex, race, socioeconomic status, current smoker, hypertension, diabetes, congestive heart failure, cerebral vascular disease, peripheral vascular disease, chronic kidney disease, and time since non-ACS hospitalization.

†Adjusted for covariates as above with time since last ACS hospitalization in place of time since non-ACS hospitalization.

‡ p < 0.05 compared to < 6 months.

§ p < 0.01 compared to < 6 months.

|| p < 0.001 compared to < 6 months.

## Discussion

In our cohort, recent non-ACS hospitalization was not associated with a substantial difference in preventive medication use in patients with known CAD. Non-ACS hospitalizations represent a potential missed opportunity to improve preventive care of patients with CAD. This is evidenced by the 10-20% increase in use of preventive medications among patients recently hospitalized for ACS.

Since 2003, more than 90% of patients treated within the VA health care system were discharged on preventive medications after ACS [4]. The quality measures associated with this success do not currently apply to CAD patients with non-ACS hospitalizations [1-3]. This is of particular importance in light of the proportion of patients admitted for non-ACS diagnoses prior to the index event. While less than 25% of our cohort had been hospitalized for ACS during the past 2 years, nearly 50% had been hospitalized for other diagnoses in that period. Targeting hospitalization as an opportunity to improve preventive care has been demonstrated in efforts to increase influenza and pneumococcal vaccination [16-18]. A similar approach targeting all hospitalized CAD patients has the potential to increase the proportion of patients who receive preventive medications and thereby prevent recurrent ACS events.

The importance of utilizing all care episodes to improve comprehensive patient care is underscored by recent national health legislation that supports accountable care organizations whereby providers are jointly held accountable for achieving quality improvement measures and reducing the rate of spending growth [19-21]. Optimizing preventive medications on discharge after non-ACS hospitalizations would potentially impact providers and systems by reducing hospitalizations that would be uncompensated in an accountable care model. In addition, initiation of preventive CAD medications in patients after non-ACS hospitalizations may increase the proportion of patients achieving proposed quality measures such as goal cholesterol levels [22].

Despite 90% of patients being prescribed preventive medications on discharge for ACS, we observed only 68 to 82% of eligible patients were taking individual preventive medications 6 months after ACS. This is consistent with prior studies that have demonstrated medication nonadherance is common after discharge for ACS [23-29]. Although prescribing medications for CAD after non-ACS hospitalizations would increase the proportion of patients provided the opportunity to take cardioprotective medications, reducing patient non-adherence to these medications remains a significant barrier to optimal risk reduction.

The benefit of preventive medications is influenced by the risk of coronary events in follow-up. Furthermore, in some patients the benefit of preventive therapies may be mitigated by comorbid conditions such as dementia or cancer. We conducted several sensitivity analyses to minimize the possibility that the observed effects of time since ACS or non-ACS hospitalization on preventive medication use were influenced by these factors. Exclusion of patients with dementia or cancer did not influence the effect size of time since ACS hospitalization or non-ACS hospitalization. In our primary analysis, we included patients with a history of coronary atherosclerosis but no history of myocardial infarction or unstable angina. This may have resulted in the inclusion of patients with coronary disease at lower risk of ischemic events. Stratification of our analysis according to history of past myocardial infarction did not influence the effect size of time since ACS hospitalization or non-ACS hospitalization. This suggests the observed association is unlikely to merely a function of including low-risk patients with a remote history of a coronary event. While the long term benefit of β-blockers after ACS or the benefit of ACE-I or ARB in CAD patients at lower risk of recurrent events is debated,[30] the stability of our findings across all medication classes suggest an important effect of time since ACS hospitalization on preventive medication use that is not apparent with non-ACS hospitalization.

Strengths of our study include 100% inclusion of patients with AMI, large sample size, geographic diversity, and the ability to assess the impact of prior ACS and non-ACS care episodes on preventive care. Our study has several limitations. First, the time interval since last hospitalization was determined from previous admissions to VA health care. As a result, there is a potential for misclassification of time since hospitalization for patients previously hospitalized in non-VA settings. If patients recently admitted to non-VA hospitals for ACS are also more likely to be prescribed preventive medications, this misclassification would be expected to bias the observed effect of recent ACS hospitalization toward the null. Second, there is potential for misclassification of aspirin use related to the ability to obtain aspirin without a prescription. The similar effect of time since discharge on prescription of all classes of secondary preventive medications provides reassurance this did not overly influence our findings. Third, the CCFCS data registry does not separate statin medications from other lipid lowering therapies. Studies of lipid lowering therapy in secondary prevention of CAD have focused on statin medications and our results may overestimate the proportion of patients on lipid lowering therapy with greatest evidence of benefit. Fourth, our use of administrative data may have led to misclassification of past CAD history. We were inclusive of a range of ICD-9 codes for coronary disease in our main analysis, but restriction to history of myocardial infarction suggests this did not overly influence our results. Fifth, our study is limited to patients of VA medical centers. Previous studies have demonstrated patients receiving care within the VA receive recommended preventive care more often than patients covered by other health care systems [31,32]. Studies to update knowledge on remaining gaps in secondary prevention of CAD in non-VA settings should be considered. Sixth, our cohort of CAD patients may have included those with a myocardial infarction secondary to either increased oxygen demand or reduced supply as opposed to acute plaque rupture [33]. Although this type of myocardial infarction is less common,[6] the relative benefit to secondary preventive medications for CAD in these patients is unclear. Further, our exclusion criteria for use of preventive medications were dependent on administrative diagnostic codes, however we were intentionally broad in our exclusion criteria to ensure all patients were reasonable candidates for secondary preventive medications. Finally, our analysis did not include covariates for other processes of care that may influence use of preventive medications. Prior studies have suggested early outpatient follow-up after ACS [28] and subspecialty care [4] may influence use of preventive medications. Understanding the interplay of all care processes may help in designing best strategies to improve preventive care for CAD.

## Conclusion

Preventive medications for coronary artery disease are critically important in reducing ischemic events and death. While significant strides have been made to improve the provision of these medications at discharge after ACS, our analysis suggests this strategy fails to optimize the preventive care of patients discharged for non-coronary events. Consideration should be given to expanding the scope of preventive measures currently targeted at discharge for ACS to all care episodes for patients with coronary disease.

## List of abbreviations

CAD: coronary artery disease; ACS: acute coronary syndrome; ACE-I: angiotensin-converting enzyme inhibitor; ARB: angiotensin II receptor blocker; CCFCS: Cardiac Care Follow-Up Clinical Study; ICD-9: International Classification of Diseases, Ninth Revision; VA: Veterans Affairs.

## Competing interests

The authors declare that they have no competing interests.

## Authors' contributions

SMB conceived and designed the study, completed the statistical analysis, and drafted the manuscript. CLB participated in the design of the study, the completion of the statistical analysis, and helped to draft the manuscript. CM participated in analysis and interpretation of the data and revised the manuscript critically for important intellectual content. TMM participated in the design of the study, interpretation of the data, and revising the manuscript for important intellectual content. SDF participated in the study design, analysis and interpretation of the data, and helped to draft the manuscript. The authors declare that they have no competing interests and all authors read and approved the final manuscript.

## Pre-publication history

The pre-publication history for this paper can be accessed here:

http://www.biomedcentral.com/1471-2261/11/42/prepub
